# Smile Esthetics and Rotated Maxillary Lateral Incisors

**DOI:** 10.1155/ijod/4968959

**Published:** 2026-02-04

**Authors:** Alexa Spokane, Daniel Rinchuse, Thomas Zullo, John Burnheimer

**Affiliations:** ^1^ Department of Advanced Education in Orthodontics and Dentofacial Orthopedics, Seton Hill University, Greensburg, Pennsylvania, USA, setonhill.edu

## Abstract

**Introduction:**

Although tooth rotations are a common orthodontic problem, little research has addressed how lay observers perceive these rotations. Consequently, the aim of this study is to determine laypersons’ esthetic perceptions of changes in the rotation of the maxillary lateral incisors.

**Materials and Methods:**

An intraoral digital scan of a female adult’s dentition was manipulated with ClinCheck software to create four smile conditions: (A) no rotation either maxillary lateral incisor, (B) 20° mesial‐out rotation of both, (C) 30° mesial‐out rotation, and (D) 40° mesial‐out rotation. Each condition was shown with frontal and three‐quarter view images. A panel of laypeople (age 18–30, no dental training) rated the attractiveness of each smile using a 100 mm visual analog scale (VAS; 0 = least esthetic, 100 = most esthetic). The score for each condition was recorded. A mixed‐model two‐way ANOVA (gender × rotation) with repeated measures was used to analyze the data.

**Results:**

No‐rotation smiles were rated significantly more attractive than any smiles with rotated lateral incisors (*p*  < 0.05). Smiles with a 40° rotation received the lowest esthetic scores and were significantly less attractive than the others (*p*  < 0.001). Laypersons did not significantly distinguish between 20° and 30° rotations (*p*  > 0.05). There was no significant difference in ratings between male and female panelists (*p* = 0.104). No interaction effect between panelist gender and rotation level was observed.

**Conclusion:**

Laypeople preferred the smile with no rotation of the lateral incisors. They could not perceive a notable esthetic difference between 20° and 30° rotations. However, a 40° rotation was perceived as substantially less esthetic. Panelist gender did not affect esthetic ratings.

## 1. Introduction

Orthodontic treatment outcomes are closely tied to esthetics and societal perceptions of attractiveness [1–8]. The definition of attractiveness evolves over time and is largely influenced by social norms and the “beauty culture” established by laypeople [9–11]. Although orthodontists’ esthetic judgments have traditionally been held in high regard, there has been a shift toward a more patient‐ centered approach that emphasizes patient autonomy in esthetic decisions [12, 13].

Patients often seek orthodontic treatment with the goal of improving their appearance [6, 8]. Physical attractiveness has a profound influence on many aspects of life. It affects not only how individuals perceive themselves, but also how others perceive them [1, 3, 5, 7]. People deemed attractive are frequently perceived as having more positive personal qualities and tend to experience more successful social and professional lives than those considered unattractive [3]. Although beauty standards change over time, their influence on life decisions remains constant [1–6]. A major esthetic focus in orthodontics is the maxillary anterior dentition, given its high visibility during speech and smiling [14–16].

According to Ricketts [17], the “golden proportion” defines optimal anterior tooth display: the visible width of the maxillary lateral incisor should be about 62% of the width of the central incisor, and the visible width of the canine about 62% of the lateral incisor, when viewed from the front. Clinicians have often used these divine proportions as a guideline for smile esthetics, even though the golden proportion concept is not evidence‐based [17–19]. The golden proportion as a reference standard was utilized for this investigation, acknowledging its limitations. If an individual’s tooth dimensions adhere to the golden proportion, the smile is generally perceived as more attractive by both dental professionals and laypeople. However, when a tooth is rotated out of its proper position in the arch, the ideal inter‐tooth width ratios are disrupted [18].

Treatment decisions in orthodontics have typically been driven by the clinician’s expertise, but modern philosophies encourage consideration of the patient’s esthetic preferences in the treatment plan [12, 13]. It is, therefore, important to study and understand laypeople’s esthetic perceptions [19, 20]. Laypeople tend to be more tolerant than dental professionals of small deviations in lateral incisor alignment [21–26]. With the current emphasis on patient‐centered care, it is essential to investigate laypeople’s perceptions of rotated teeth [1].

Although tooth rotations are a common orthodontic malocclusion, little research has addressed how lay observers perceive these rotations. Much of the existing literature on smile esthetics has focused on crown width‐to‐length ratios and other microesthetic parameters (for example, modifications of the golden proportion), rather than the impact of tooth rotation on perceived attractiveness [27–29]. The aim of this study is to determine how laypersons perceive changes in the degree of rotation of the maxillary lateral incisors.

## 2. Materials and Methods

This observational cross‐sectional study was conducted at the Center for Orthodontics, Seton Hill University, Greensburg, Pennsylvania and approved by the Seton Hill University Institutional Review Board (IRB approval # SP24‐06). To represent the adult demographic most interested in orthodontic treatment, a female model between 20 and 30 years of age was selected, as this age group has been shown to have a more favorable view of orthodontic treatment [30]. The model met the inclusion criteria of a consonant smile arc and no notable rotations, discolorations, or malocclusions. Her maxillary anterior teeth fell within the approximate divine proportions described by Ricketts [17, 18]. Exclusion criteria for the model included a severe overbite or overjet, any significant dental rotations, and any dental or soft tissue asymmetry. The model provided informed consent and granted permission for her digital dental scan to be used and modified for research purposes.

An iTero digital intraoral scan (Align Technology, San Jose, CA) of the model’s maxillary and mandibular arches was obtained. To minimize confounding variables, only the teeth and gingiva were shown in the images. Radlanski et al. [31] determined that when only the anterior teeth are visible, observers cannot discern the person’s sex.

For this study, mesial‐out rotations of the maxillary lateral incisors were studied. The methods followed approaches similar to those of Bukhary et al. [32], Oliveira et al. [33], and Yang et al. [34]. Using the Invisalign ClinCheck software, the digital dental model was manipulated to produce four different smile conditions (Figure 1): (A) the maxillary lateral incisors with no rotation (ideal alignment); (B) 20° mesial‐out rotation; (C) 30° mesial‐out rotation; (D) 40° mesial‐out rotation. A pilot test with orthodontic residents indicated that a 10° rotation was not readily perceptible and was excluded from the study. Each condition was presented using two images of the smile (one frontal view and one three‐quarters view) displayed side by side. Presenting both views helped viewers understand that the size of the tooth was constant and only its rotation changed [35].

Figure 1Digital scans illustrating degrees of maxillary lateral incisor rotation. (A) Ideal alignment, (B) 20° mesial‐out, (C) 30° mesial‐out, and (D) 40° mesial‐out.

**Figure:**
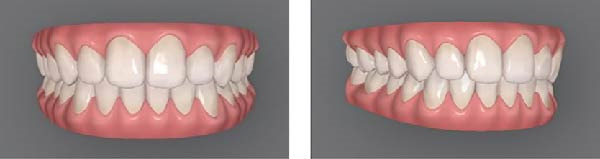
(A)

**Figure:**
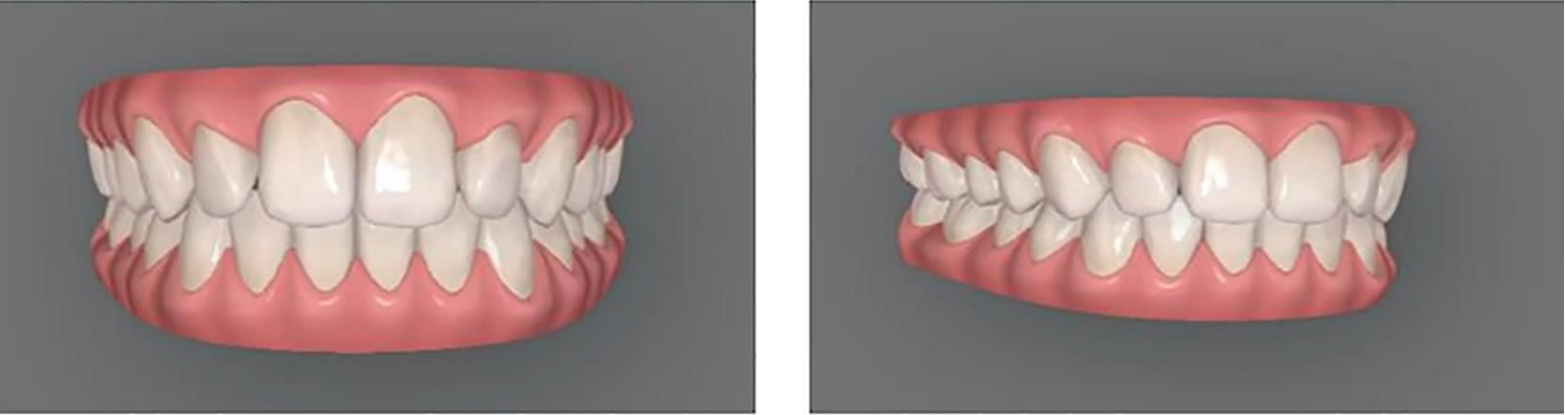
(B)

**Figure:**
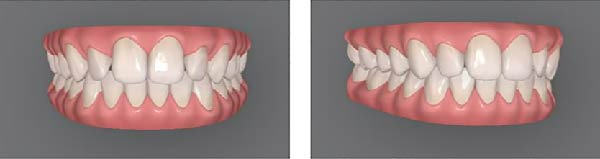
(C)

**Figure:**
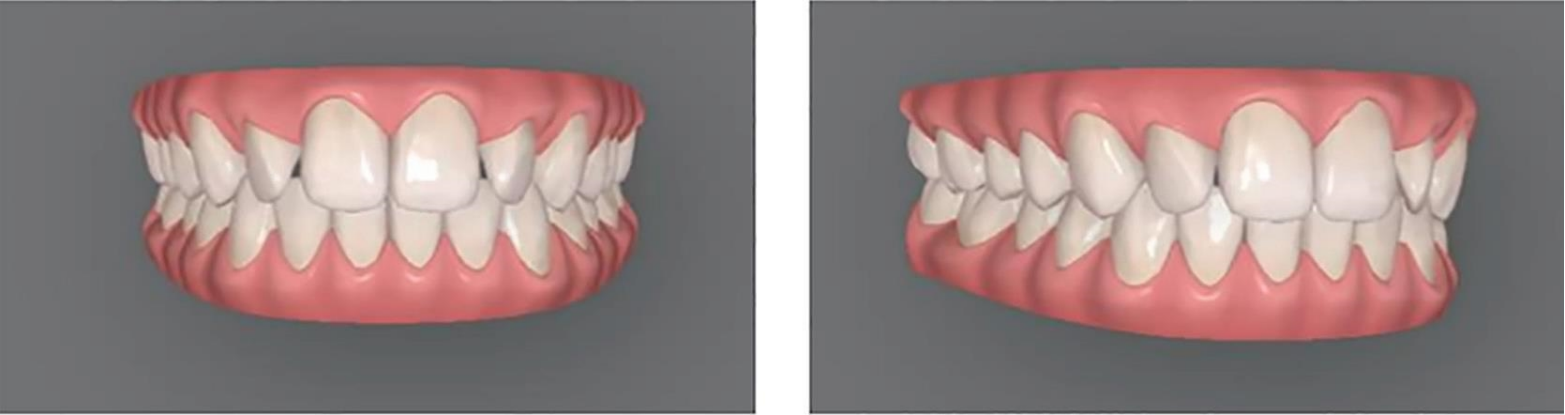
(D)

All survey materials, including instructions and images, were uploaded to an online survey platform (SurveyMonkey, https://www.surveymonkey.com). The survey collected basic demographics and then presented the four smile conditions in random order. An unmarked 100 mm visual analog scale (VAS) [36] was used beneath each image pair to record the panelist’s esthetic rating. Once a panelist submitted a rating for a given image, it could not be changed. There was no overall time limit for the survey. The participants were not informed of the specific aims or hypotheses of the study.

The sample consisted of laypersons between 18 and 30 years old with no education or work experience in dentistry or orthodontics. Panelists were recruited through the SurveyMonkey audience platform. Whitesides et al. [37] noted that adults in the 18–30 age range have the highest odds of recent orthodontic visits, making this group particularly relevant for studying perceptions of orthodontic outcomes. Restricting the sample to this young adult group also helped reduce variability in responses that might occur across a broader age range. Demographic information was collected at the beginning of the survey to confirm inclusion criteria.

A total of 221 individuals accessed the survey. Five respondents were excluded because they indicated “Do not wish to participate,” leaving 216 who provided usable responses. The distribution of self‐reported gender among these 216 respondents is shown in Table 1. Given the very small numbers of transgender and nonbinary, these were omitted from further statistical analysis. The final sample consisted of 207 participants classified as male or female. Among those, complete survey data (ratings for all four conditions) were available for 117 participants (42 male, 75 female). These 117 responses were used for the primary statistical analysis.

**Table 1:** Distribution of gender.

Valid	Frequency	Percent	Valid percent	Cumulative percent
Male	85	39.4	39.4	39.4
Female	122	56.5	56.5	95.8
Transgender	6	2.8	2.8	98.6
Non‐binary	3	1.4	1.4	100.0
Total	216	100.0	100.0	—

Data were analyzed using IBM SPSS Statistics (Version 29.0; IBM Corp). A two‐factor mixed ANOVA was performed with gender (male and female) as a between‐subjects factor and rotation (0°, 20°, 30°, and 40°) as a within‐subject (repeated measures) factor. Pairwise comparisons between rotation levels were conducted with Bonferroni adjustments. Statistical significance was set at *p*  < 0.05 for all tests.

## 3. Results

The gender of the rater had no significant effect on the esthetic ratings (*F* = 2.69, df = 1,116, *p* = 0.104; Table 2). On average, male panelists gave slightly higher attractiveness scores than female panelists, but this difference was not statistically significant.

**Table 2:** Main effects of gender.

Gender	Mean	Std. error	Lower CI	Upper CI
Male	61.884	3.093	55.757	68.011
Female	55.517	2.342	50.877	60.156

*Note: F* = 2.69; df = 1116; *p* = 0.104. The effect of gender on perceived attractiveness as measured utilizing the visual analog scale.

Abbreviation: CI, 95% confidence interval.

In contrast, the degree of lateral incisor rotation had a highly significant effect on perceived attractiveness (*F* = 35.796; df = 3,348; *p*  < 0.001; Table 3). Pairwise comparisons among the four rotation conditions (Table 4) showed that all comparisons were statistically significant (*p*  < 0.05) except the comparison between the 20° and 30° rotation conditions, which did not significantly differ. In other words, the smile with no rotation was rated significantly more esthetic than any of the rotated conditions (0° vs. 20°, 0° vs. 30°, and 0° vs. 40° were all *p*  < 0.05). The 40° rotation condition was rated significantly less esthetic than the other three conditions (40° vs. 0°, *p*  < 0.001; 40° vs. 20°, *p*  < 0.01; 40° vs. 30°, *p*  < 0.01). The difference in mean ratings between the 20° and 30° rotation conditions was small and not statistically significant (*p*  > 0.05).

**Table 3:** Main effects of rotation.

Rotation	Mean	Std. error	Lower CI	Upper CI
0 Rotation	72.232	2.353	67.571	76.892
20°	58.110	2.506	53.147	63.074
30°	56.378	2.385	51.654	61.103
40°	48.080	2.444	43.239	52.922

*Note: F* = 35.796; df = 3348; *p* < 0.001. The main effect of the degree of rotation and perceived attractiveness as measured utilizing the visual analog scale.

Abbreviation: CI, 95% confidence interval.

**Table 4:** Pairwise comparisons.

Rotation (*I*)	Rotation (*J*)	Mean difference (*I*−*J*)	Std. error	Sig.	95% Confidence interval for difference	
					Lower bound	Upper bound
0 Rotation	20°	14.121 ^∗^	2.518	<0.001	7.362	20.880
	30°	15.853 ^∗^	2.581	<0.001	8.926	22.781
	40°	24.151 ^∗^	2.798	<0.001	16.640	31.663
20°	0 Rotation	−14.121 ^∗^	2.518	<0.001	−20.880	−7.362
	30°	1.732	2.122	1.000	−3.963	7.427
	40°	10.030 ^∗^	2.189	<0.001	4.155	15.906
30°	0 Rotation	−15.853 ^∗^	2.581	<0.001	−22.781	−8.926
	20°	−1.732	2.122	1.000	−7.427	3.963
	40°	8.298 ^∗^	1.892	<0.001	3.219	13.377
40°	0 Rotation	−24.151 ^∗^	2.798	<0.001	−31.663	−16.640
	20°	−10.030 ^∗^	2.189	<0.001	−15.906	−4.155
	30°	−8.298 ^∗^	1.892	<0.001	−13.377	−3.219

*Note:* Pairwise comparisons among the four rotation conditions.

^∗^ *p* = 0.05

There was no significant interaction between gender and rotation (*F* = 1.344, df = 3,348, *p* = 0.260; Table 5). This indicates that the pattern of esthetic ratings across the rotation conditions was similar for male and female panelists. In summary, laypeople overwhelmingly preferred the smile with no lateral incisor rotations and gave progressively lower ratings as the severity of rotation increased, finding the 40° rotation least attractive. These trends were observed in both male and female respondents.

**Table 5:** Effects between gender and rotation.

Gender	Rotation	Mean	Std. error	Lower CI	Upper CI
Male	0 Rotation	74.023	3.752	66.592	81.455
Male	20°	61.767	3.996	53.853	69.682
Male	30°	57.930	3.803	50.397	65.463
Male	40°	53.814	3.898	46.094	61.534
Female	0 Rotation	70.440	2.841	64.813	76.067
Female	20°	54.453	3.026	48.461	60.446
Female	30°	54.827	2.880	49.123	60.530
Female	40°	42.347	2.951	36.502	48.192

*Note: F* = 1.344; df = 3,348; *p* = 0.260. The was no significant interaction between gender and rotation of the maxillary lateral incisor.

Abbreviation: CI, 95% confidence interval.

## 4. Discussion

As expected, rotation of the maxillary lateral incisors had a detrimental effect on perceived smile esthetics. In this study, the smile with no rotation of the lateral incisors (Figure 1A) received the highest esthetic ratings from lay participants (mean VAS score of 72.232 mm) and was rated significantly more attractive than any of the smiles with rotated lateral incisors. In contrast, the smile with a 40° rotation of the lateral incisors (Figure 1D) had the lowest mean esthetic rating (48.080 mm) and was judged significantly less attractive than the other conditions. These findings support the importance of proper anterior tooth alignment in achieving an esthetic smile and are consistent with the concept of the “golden proportion.” According to this concept, there are ideal tooth‐width ratios that make a smile most pleasing [17–19], and when teeth are correctly aligned, these ratios are maintained to produce optimal visual harmony [18, 19]. Rotation of an anterior tooth disrupts the golden proportion and thereby adversely affects perceived esthetics [16, 18]. A rotated lateral incisor gives the impression of a reduced mesiodistal width, even though the actual tooth size is unchanged, which likely explains why the rotated conditions were judged less attractive by the panelists [18].

Although orthodontists and trained examiners can detect relatively small deviations in tooth alignment, laypeople are generally more forgiving of minor rotations and asymmetries [21–23]. In the present study, lay participants did not perceive a notable esthetic difference between the 20° rotation (Figure 1B) and the 30° rotation (Figure 1C) of the lateral incisors; their attractiveness ratings for these two conditions were statistically equivalent. This suggests that a 20° mesial‐out rotation of a lateral incisor may not be pronounced enough for the average lay observer to distinguish from a 30° rotation in terms of smile attractiveness. From a clinical perspective, if laypeople do not view a mild‐to‐moderate rotation (in the range of 20°–30°) as making a smile less esthetic, correcting such a rotation might not greatly enhance the patient’s perceived outcome. In other words, it may not be necessary to subject a patient to orthodontic correction of a 20° rotated lateral incisor purely for esthetic reasons, especially if even a 30° rotation is not appreciably recognized as unattractive by lay observers [24–26].

On the other hand, the 40° rotation was clearly discernible to laypeople and had a significantly negative impact on esthetic ratings. Additionally, this large rotation of the maxillary incisor, if left untreated, would negatively impact the completed orthodontic result. Therefore, beginning with treatment planning and throughout treatment, practitioners should prioritize correcting severe rotations of anterior teeth, as laypeople can detect these rotations and the effect of these on perceived attractiveness. These findings reinforce that while minor rotations might remain undetected by laypersons, more pronounced rotations (on the order of 40°) are likely to be perceived negatively and should be addressed to meet patient expectations [16].

This study has several limitations [38]. First, its cross‐sectional design provides a “snapshot” of lay perceptions at one point in time. A person’s esthetic judgment could change over time or with social trends, thereby, limiting the timeliness of the results. Second, the study was conducted with respondents from a single country (the United States), and esthetic perceptions may vary in different cultures or populations; thus, the generalizability of these findings internationally may be limited. Additionally, we used a digitally created smile model rather than photographs of a real person. While this approach allowed precise control over the single variable of lateral incisor rotation, it may not capture all the nuances of how a real smile is perceived. The panelists saw only the teeth and gingiva of the model with no surrounding facial context.

This isolation of the dental features (a deliberate delimitation of the study) could have influenced the way participants judged attractiveness, since factors, like lip shape, facial symmetry, and dynamic expressions, were not represented. Finally, we intentionally altered only the degree of rotation of the lateral incisors between images. Other factors that might affect esthetics (such as tooth color, gingival display, or arch form) were held constant. While this strengthens internal validity by focusing on the rotation variable, it also means the results pertain specifically to rotation changes in an otherwise idealized smile.

This research concentrated exclusively on laypeople’s perspectives. Future studies could include both laypeople and orthodontic professionals to compare differences in esthetic assessments based on the observer’s level of dental training. Notably, prior work has shown that dental professionals and laypersons can differ in their perceptions of certain dental esthetic issues (for example, in cases of congenitally missing lateral incisors) [38]. Understanding these differences is increasingly important as we strive to balance clinical objectives with patient satisfaction in the era of patient‐centered care and informed consent.

## 5. Conclusion

Based on the esthetic ratings in this study, the following conclusions can be drawn:

• Laypeople rated the smile with no rotation of the maxillary lateral incisors as the most esthetic.
• Laypeople did not perceive a significant esthetic difference between a 20° and a 30° mesial‐out rotation.
• The smile with a 40° rotation of the maxillary lateral incisors received the lowest esthetic attractiveness rating.
• The gender of the rater had no statistically significant effect on the esthetic rating.

## Funding

No funding was received for this manuscript.

## Conflicts of Interest

The authors declare no conflicts of interest.

## Data Availability

The data that support the findings of this study are available from the corresponding author upon reasonable request.
